# High‐Throughput Contact Flow Lithography

**DOI:** 10.1002/advs.201500149

**Published:** 2015-06-24

**Authors:** Gaelle C. Le Goff, Jiseok Lee, Ankur Gupta, William Adam Hill, Patrick S. Doyle

**Affiliations:** ^1^ Department of Chemical EngineeringMassachusetts Institute of Technology77 Massachusetts AvenueCambridgeMA02139USA; ^2^ Novartis Institutes for Biomedical Research250 Massachusetts AvenueCambridgeMA02139USA; ^3^ School of Energy and Chemical EngineeringUlsan National Institute of Science and TechnologyEonyang‐eupUlju‐gunUlsan689‐798South Korea

**Keywords:** contact flow lithography, encoded particles, high‐throughput, hydrogel, microfluidics

## Abstract

**High‐throughput fabrication of graphically encoded hydrogel microparticles** is achieved by combining flow contact lithography in a multichannel microfluidic device and a high capacity 25 mm LED UV source. Production rates of chemically homogeneous particles are improved by two orders of magnitude. Additionally, the custom‐built contact lithography instrument provides an affordable solution for patterning complex microstructures on surfaces.

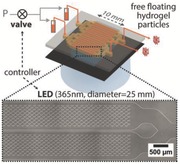

Recent advances in fabrication techniques have created new opportunities for applications of polymer particles, beyond spherical particles.^1^ Anisotropic polymer particles with precise shapes or heterogeneous chemistries, in particular anisotropic hydrogel particles, have demonstrated unique advantages in numerous fields. For drug delivery, tissue engineering, and diagnostic imaging, engineering nano and microparticles' shape is a way to tailor particle penetration and degradation properties.^2^ In the field of biosensing, unique shape and graphical patterns of particles have brought new strategies for encoding complex particle libraries for multiplex sensing applications.^3^ A common requirement to all these applications is the need for robust, affordable, and rapid techniques for particle fabrication.

Conventional methods for the fabrication of micrometer‐sized hydrogel particles, such as dispersion, precipitation, and emulsion polymerization, are often limited to the production of polydisperse suspensions of spherical particles.^4^ Similarly, droplet‐based microfluidic techniques enable high‐throughput polymer particle production but are usually restricted to spheres or spheroids. Contact photolithography and replica molding, already used to pattern polymeric structures on surfaces, have been successfully adapted to the production of nonspherical particles. Originally developed for the production of submicrometer features in the semiconductor industry,^5^ photolithography techniques use light to transfer a pattern from a photomask to a photopolymerizable material. Shape‐coded hydrogel particles in the 50–1000 μm range were successfully patterned using contact photolithography, using a photomask placed in direct contact with a layer of monomer solution.^6^ Replica molding, also known as imprint lithography,^7^ is directly inspired from the soft lithography techniques developed for the fabrication of microfluidic devices.^5^ Replica molding of particles consists of pouring a liquid monomer into a negative mold with the desired shape and dimensions, and photocrosslinking the material in the mold. Nevertheless, both techniques are static batch processes with limited throughputs and particle collection time and set‐up times in between runs often reduce the synthesis rates.

The development of the flow‐photolithography technique enabled significant progress toward automation and scale‐up of microparticle synthesis using microfluidic channels.^8^ Particles are synthesized inside a polydimethylsiloxane (PDMS) microfluidic channel filled with a photocurable monomer solution, using microscope‐based illumination and automated control of exposure to ultraviolet (UV) light. Where exposed to UV light, the monomer crosslinks and solidifies into a microparticle. Due to PDMS permeability to oxygen, oxygen is present at high concentration near the PDMS channel walls and locally inhibits the free‐radical polymerization. This inhibition creates a thin lubrication layer of uncured monomer (typically 2.5 μm‐thick) at the top and bottom sides of the channel and results in free‐floating particles that can be transported through the channel with the stream of monomer.^9^ Particles are collected in an outlet reservoir while the polymerization process is repeated inside the channel. The method was demonstrated on polyethylene glycol diacrylate (PEGDA) hydrogels, but is applicable to any free radical polymerization reaction.^9^, ^10^ Several research groups successfully applied flow lithography to synthesize particles with complex graphical codes based on shapes,^11^ 1D‐barcodes,^12^ or even 2D‐barcodes.^13^ Recent studies also investigated 3D‐particle patterning.^14^

The technique was initially proposed by Dendukuri et al. as continuous flow lithography (CFL), with sequential UV pulses sent through the photomask on a continuous flow of monomer.^15^ This process was however limited in resolution at high flow rates, since the polymerizing particles moved significantly during exposure, resulting in blurred particles. In the next iteration of the technique, stop‐flow lithography (SFL), photopolymerization was performed in a stationary monomer, optimizing the patterning resolution. In addition, much higher flow rates could be used to flush particles out of the channel. As a result, both particle resolution (10–100 μm) and synthesis throughput (10^4^ per hour) were enhanced compared to CFL.^8^

While the conventional microscope‐based flow lithography brings multiple advantages, such as intense light power surface density through the objective, fine resolution, and control over focal adjustment, it critically limits the illumination area and significantly decreases the number of particles that can be synthesized in a single exposure. Typically, the homogenous illumination area with a 20× objective is less than 500 μm in diameter, which severely limits the number of particles per exposure and the particle synthesis rate. Moreover, the cost of the microscope instrument and objective hinder the possibility of using multiple parallel synthesis setups in terms of industrial scale up.

To overcome the above limitations of CFL and SFL, we designed a novel bench‐top contact flow lithography system, with versatile lithography functions, and we successfully achieved particle synthesis at ultrahigh throughput. With our customized low cost contact photolithography system providing strong and homogeneous illumination across 23 mm and rationally designed microfluidic channels, we dramatically increased the particle synthesis rate by two orders of magnitude (>10^6^ 100 μm sized particles per hour) while maintaining excellent particle resolution and homogeneous physicochemical property of particles. Furthermore, the use of this cost‐efficient platform can be easily extended to a variety of photolithography applications.

The investigated contact flow lithography station is composed of three major parts (**Figure** **1**a, from bottom to top): an illumination unit triggering microparticle photopolymerization, a stage unit holding the microfluidic device, and an imaging unit (charged‐coupled device (CCD) based camera) enabling to align the mask with the microfluidic device. To build the illumination unit, a high power UV light‐emitting diode (LED) light source (365 nm, 700 mA) was collimated into a 25 mm beam using an aspheric condenser lens without diffuser and assembled to a precision XYZ‐rotation stage. A specially designed photomask adapter for 25 mm chrome masks was 3D‐printed and tightened to the UV illumination unit. The stage unit was fixed and fastened to a damped post to inhibit vibration from the solenoid valve during microparticle synthesis. Finally, the imaging unit was built into another XYZ linear translation stage for precision motion. In this manner, we are able to independently control the position of both the imaging unit and the illumination unit with regard to the stage unit. Before polymerization, the photomask is placed in contact with the UV transparent device to be patterned. Minimizing the distance between the mask and the microfluidic device decreases diffraction and aberration of the UV light. In the case of microscope‐based lithography, accurate positioning of the channel in the objective focal plane was critical for particle resolution and the objective depth of field was limiting the particle thickness. For the contact lithography instrument however, collimation of the UV LED light into a straight beam is the key to well‐resolved particles with straight edges.

**Figure 1:**
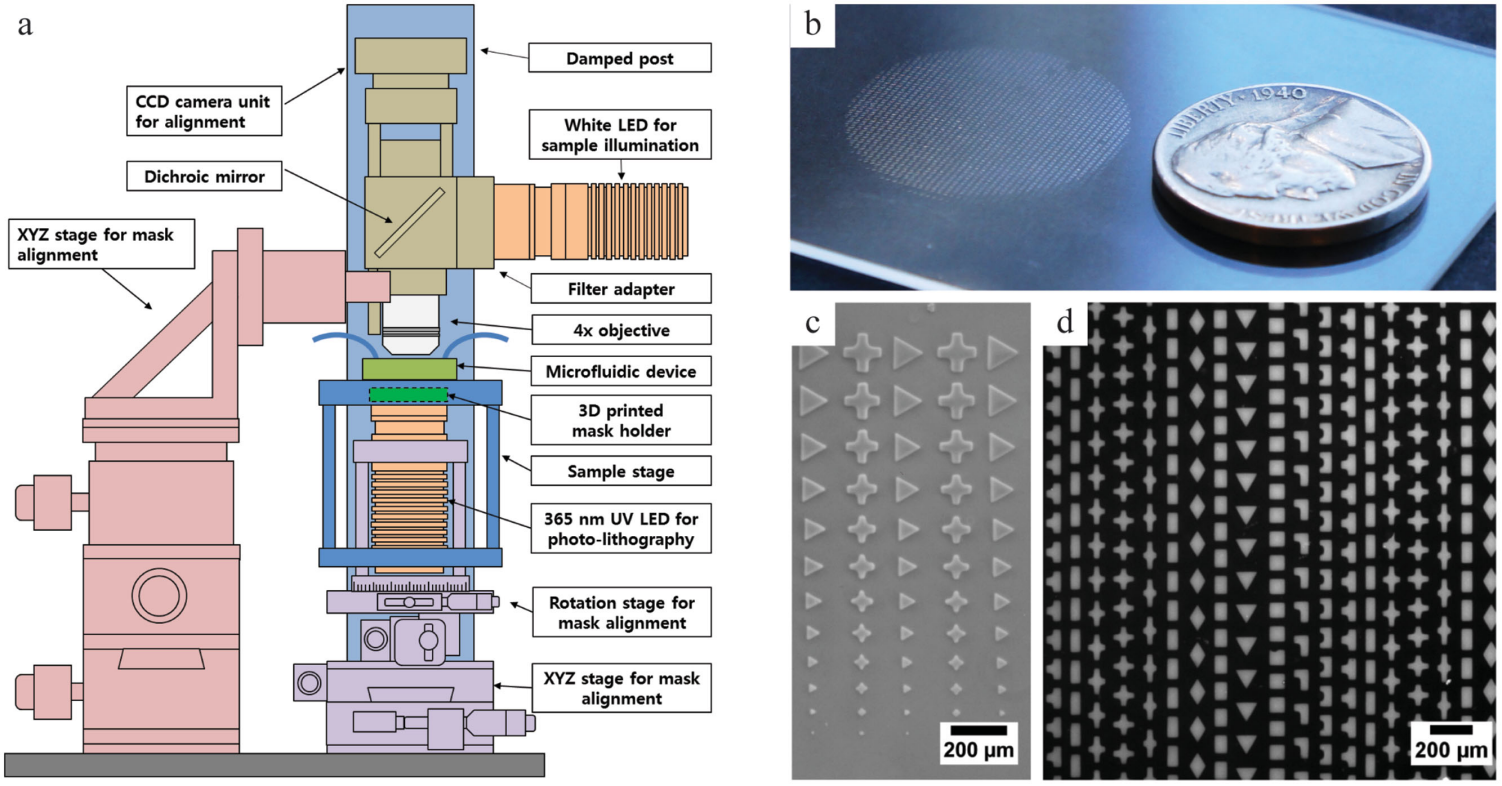
Contact lithography instrument. a) Schematic diagram of the contact lithography system. b) Photograph showing the extent of the polymerization area on glass slide (25 mm). c) Bright field microscopy image of various sized microstructure and d) fluorescence microscopy image of rhodamine‐B labeled microstructures with multiple shapes on a glass slide.

In order to investigate the homogeneity of the illumination provided by the LED source, we polymerized an array of PEG microstructures with various shapes on a glass substrate across the entire beam of 25 mm diameter (Figure 1b). The size and overall shape of polymerized structures appeared in excellent agreement with the mask pattern, with sharp edges and straight side walls. Structure quality decreased only in a 1 mm peripheral zone, where underpolymerization was observed (Figure S3, Supporting Information), resulting in a 23 mm effective area for reproducible patterning. Figure 1c demonstrates successful patterning of structures of decreasing sizes, from 140 to 20 μm. Incorporating rhodamine‐B in the monomer solution and analyzing the fluorescent signal of the labeled microstructures ensured that the synthesized microstructures were not only physically but also chemically homogeneous, as shown in Figure 1d (coefficient of variation (CV) = 6.8%). The power density of the collimated LED beam was 125 mW cm^−2^, which is about three times lower than the value measured on our standard microscope‐based SFL system. Therefore, we increased UV exposure times from 70 to 200 ms to achieve similar chemical conversion for 100 μm sized particles.

Flow lithography protocols were successfully developed for the contact flow lithography system, enabling the synthesis of chemically and physically homogeneous hydrogel particles with synthesis rates enhanced by two orders of magnitude. **Figure** **2**a describes the workflow for particle synthesis using contact flow lithography. The microfluidic device is secured on the fixed lithography stage (Figure S1, Supporting Information). The chrome photomask with the desired pattern is placed in the 3D‐printed mask holder and carefully elevated until in close proximity to the bottom of the microfluidic device, so that both the microchannel and the mask pattern can be observed simultaneously with the CCD camera. The position of the illumination unit is then adjusted to align the photomask with the microchannel (Figure S2, Supporting Information). Once aligned, the light source is elevated again until the chrome mask is in contact with the bottom of the microfluidic device. The device inlet is connected to a pressured monomer reservoir, and the outlet to a particle collection vial. The device is initially primed with monomer solution. From then, the stop‐flow lithography cycle can be decomposed in three steps. First, particles are polymerized with ≈200 ms UV exposure followed by a brief hold (≈250 ms) to ensure complete polymerization (Figure 2b). Particles polymerize locally where the UV light reaches the monomer layer and the photomask pattern is transferred as a negative to the monomer layer. Second, the monomer flow is switched on again and particles are flushed out by flowing monomer solution for a few seconds through the microfluidic channel (Figure 2c). Third, the flow of monomer is stopped for the next round of polymerization. Because of PDMS elasticity, a minimum response time is required to observe complete flow stoppage.^16^ The pressure imposed at the inlet induces a deformation of the channel top wall. When this constraint is released, PDMS relaxation creates an opposite squeeze‐flow (Figure S3, Supporting Information), requiring additional seconds to reach a complete fluid stoppage and to ensure high patterning resolution. A compressed‐air flow control system and a solenoid valve control the pressure‐driven flow of monomer inside the device. Both the valve and the LED are computer‐controlled and synchronized, making the particle production a fully automated process.^17^

**Figure 2:**
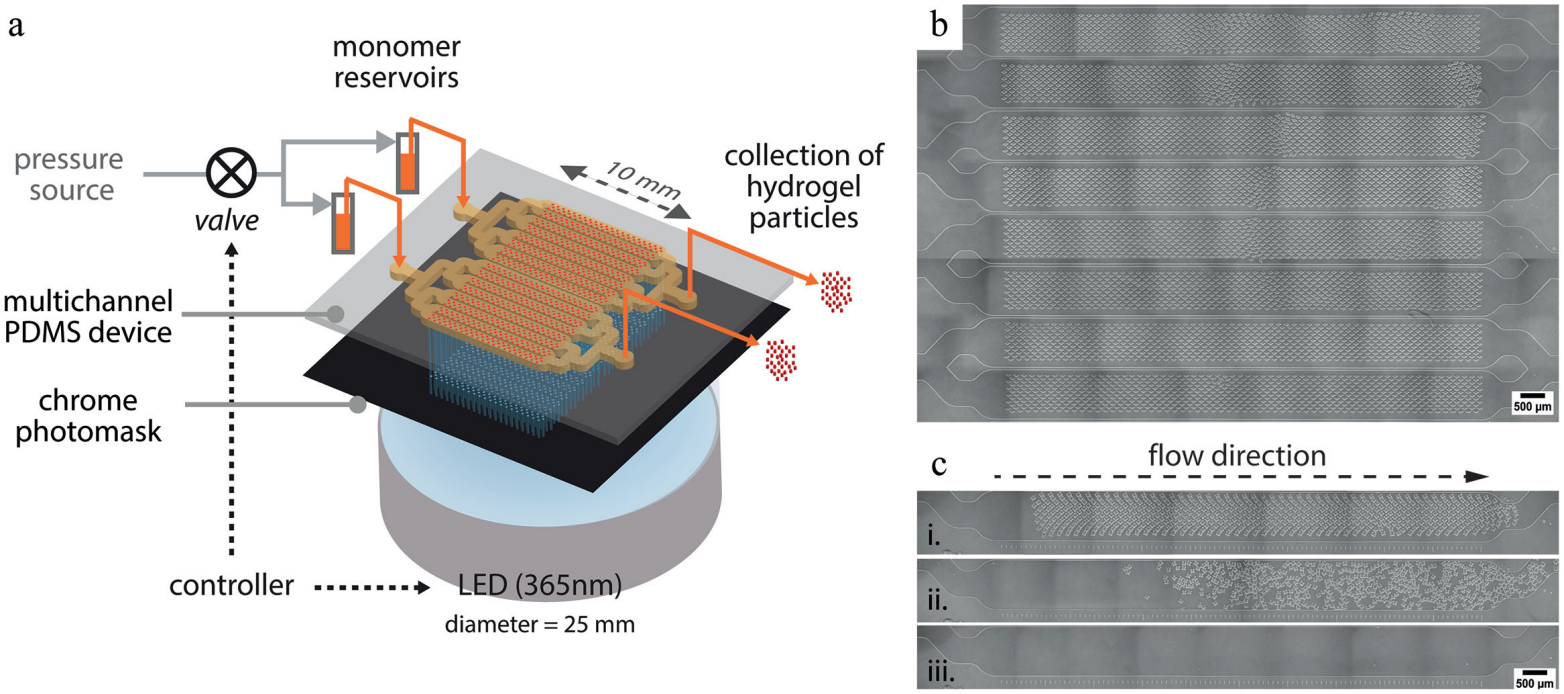
Synthesis of microparticles in flow. a) Schematic diagram describing the workflow for stop‐flow contact lithography. b) Bright field microscopy image of an entire eight‐channel module filled with diamond‐shaped particles (5760 particles) after UV exposure. c) Sequential views of particles being flushed out of the channel (*W* = 950 μm; *L* = 10 mm).

Our contact flow lithography system provides a polymerization area ≈2000 fold larger than the microscope‐based system. To take advantage of this dramatically increased illumination area, we rationally redesigned the PDMS device to integrate multiple parallel synthesis channels. We tailored the channels layout and dimensions to maximize the particle synthesis rate.

As shown in Equation (1), the particle synthesis rate depends not only on the number of particles polymerized per UV pulse (*n*_p_) but also on the duration of each cycle step: polymerizing (*t*_pol_), flushing particles out (*t*_flow_), and stopping the flow (*t*_stop_)

(1) $$\mathrm{synthesis}\;\;\mathrm{rate}=\frac{n_{p}}{t_{\mathrm{pol}}+t_{\mathrm{flow}}+t_{\mathrm{stop}}}$$

The microchannel dimensions (length L, width W, and height H) critically impact three of these parameters, namely, *n*_p_, *t*_flow_, and *t*_stop_. Theoretical analysis of a model single straight channel led to the scaling law given in Equation (2) (detailed analysis available in the Supporting Information), where *μ* represents the dynamic viscosity of the monomer fluid, *E* the elastic modulus of PDMS, and Δ*P* the pressure drop across the channel

(2) $$\mathrm{synthesis}\;\;\mathrm{rate}\approx \frac{1}{\frac{4\mu L}{EH^{2}}\left(\frac{1}{\frac{\Delta PW}{E}+\frac{H}{3}}+\frac{3E}{\Delta PW}\right)}$$

According to Equation (2), increasing the channel width or height leads to an increase in synthesis rate. We chose a channel height *H* of 50 μm in order to produce particles with 45 μm in height. The *W*/*H* aspect ratio was limited by fabrication constraints. Indeed, for *W*/*H* > 20 (*W* > 1 mm), the top wall of the PDMS channel sags. PDMS delamination under high pressure imposes an additional practical limits on the pressure imposed at the inlet.

With all other parameters fixed, Equation (2) shows that increasing the channel length tends to decrease the rate of particle synthesis. Indeed, longer channels increase both the particle flushing time and the flow stoppage time. Therefore, designs involving multiple short channels are preferable to a long serpentine channel. Contiguous parallel channels enable to maximize the coverage of the polymerization zone. As individual inlets and outlets would generate important dead space and excessive tubing, channels were grouped using with a splitting design. From a single inlet, the monomer flow was equally split into eight identical channels, using a design optimized through simulations (Figure S5, Supporting Information). Individual channel width was 950 μm and parallel channels were separated by 50 μm PDMS walls. With a channel length of 10 mm, two of these 8‐channel modules can be run side by side, covering a 16 × 10 mm polymerization zone. For microfluidic layouts with shorter channel length, the dead space occupied by the splitting flow modules upstream and downstream of the straight channels becomes too important relative to the effective particle polymerization module to be beneficial.

Particles were successfully polymerized at high volume fractions in channels (>50%) without jamming, using high density mask patterns (Movie S1, Supporting Information). Typically features on the photomask were spaced from one another by at least 25 μm and from the channel wall by at least 50 μm. It should be noted though that, at such high particle density, the flow stoppage is critical, as a residual flow may cause particles to overlap during polymerization and clog the PDMS channel. As an example, with these dimensions, up to 720 diamond shaped‐particles (≈75 μm) fitted in a unit channel, leading to 11 520 particles polymerized per exposure in the 16‐channel device. At 8 psi, complete synthesis cycles were successfully run in 7.5 s (*t*_pol_ 0.5 s; *t*_flow_ 4.5 s; *t*_stop_ 2.5 s), leading 5.6 × 10^6^ particles h^−1^ (Movie S1, Supporting Information). This represents an increase in synthesis rate of two orders of magnitude compared to the microscope‐based stop‐flow lithography system. Although the extended dimensions of the microfluidic device require a polymerization cycle time ten‐fold longer than for microscope‐based flow lithography, the dramatic increase in the number of particles produced per UV pulse leads overall to a significant 100‐fold increase in synthesis rate.

To assess the reproducibility of particle size, shape, and composition, a test panel of 12 shapes (≈75 μm) with distinctive aspect ratio and solidity was polymerized from a fluorescent monomer (Movie S2, Supporting Information). Table S1 (Supporting Information) summarizes the characteristics of the collected particles. All particles had sharp edges and straight side walls, and were flat (**Figure** **3**). The median particle thickness was 44.8 ± 1.5 μm (CV = 3.3%), when the expected value was 45 μm (50 μm‐thick channel with top and bottom 2.5 μm‐thick oxygen inhibition layers).

**Figure 3:**
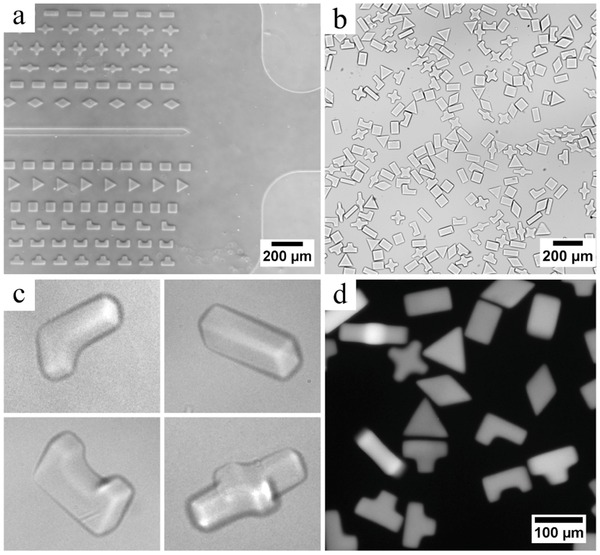
Bright field microscopy image of PEGDA hydrogel particles with various shapes a) after UV exposure, b) after collection, c) magnified view (particles are 45 μm‐thick). d) Fluorescence microscopy image of rhodamine‐B labeled PEGDA hydrogel particles.

For contact lithography, a 1:1 ratio between the photomask feature size and the particle size should be observed if the light source is perfectly collimated. The dimensions of the collected particles were in excellent agreement with mask feature size (from 98% to 106% for four shapes) and high reproducibility was demonstrated (CV = 3.6%).

Regarding particle composition, the variation of the median fluorescence intensity was found to be 7.7% (across 108 particles). Furthermore, as such encoded hydrogel particles are typically engaged in biosensing experiments,[[qv: 13a]] we demonstrated the particle porosity and functionality by incorporating a biotinylated probe inside the gel material and validating the diffusion and capture of streptavidin–phycoerythrin molecules in the gel (data not shown). In addition to polyethylene glycol diacrylate aforementioned, the method can be extended to other photopolymerizable monomers as well, and was also demonstrated on polyurethane acrylate (Movie S3, Supporting Information).

By revisiting our approach for lithography of particles, the design of our microfluidic device and the UV source, we managed to achieve very high synthesis rate and high reproducibility while working around clogging issues typical of particle suspensions at such high volume fractions. This manufacturing throughput combined with the low‐cost instrumentation paves the way for studies needing substantial numbers of particles; such as drug formulation, rheology, and 3D printing of custom suspensions. Besides the manufacturing of large quantities of particles per se for downstream applications, our system also offers a platform for fundamental studies of suspensions of complex microparticles with precise initial conditions. Indeed, it is possible to create large numbers of suspensions of arbitrarily shaped particles at high volume fractions in situ inside a microchannel, while precisely controlling the initial conditions, the material chemical and physical properties, the volume fraction, as well as the respective positioning of particles. Potential applications of interest include understanding of complex suspension dynamics,^18^ particle trajectories in microfluidic devices,^19^ particle jamming, or printing of unconventional materials.^20^ For example, **Figure** **4** presents a study of jamming of particles in a microfluidic channel. An array of stiff polyurethane acrylate particles was photopolymerized inside a channel displaying a narrow constriction at its end. When flown through the constriction, particles progressively jam. The initial conditions of the suspension can easily be varied, in order to compare their respective influence on the suspension dynamics and the jamming event.

**Figure 4:**
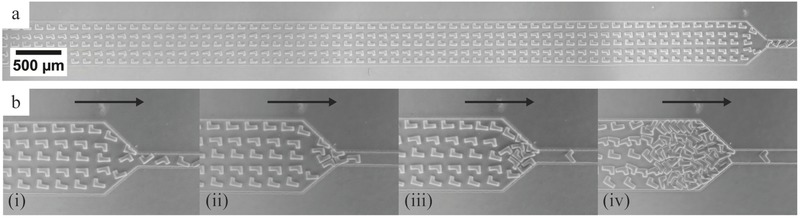
Observation of a suspension of polyurethane acrylate particles jamming at a narrow channel constriction (channel height is 35 μm). An array of precisely shaped and positioned particles was polymerized in situ inside a microfluidic channel. Under flow (direction indicated by black arrow), the rigid free‐floating particles travel toward the channel outlet and progressively jam near the constriction.

Finally, beyond particle synthesis, the contact flow lithography instrument offers versatile lithography applications and is in particular a cost‐efficient solution for patterning well‐resolved 10–1000 μm microstructures on surfaces, as previously mentioned (Figure 1c,d). Patterned hydrogel microstructures have raised notable interest in the fields of biosensing,^21^ cell culture,^22^ and cell imaging (for example, neural stem cells^23^ and spheroids^24^. Various techniques were reported for fabricating hydrogel microwells in the 100–1000 μm range: replica molding of photocrosslinkable chitosan,^24^ PEG/heparin multilayered structures,^25^ or peptide‐based gels,^26^ soft embossing of PEG gels,^23^ as well as contact photolithography to pattern PEG‐based materials.^22^ There is a need for techniques enabling to pattern multiple functional materials with high spatial control. For example, contact lithography methods were used to pattern multiple solutions containing cells and extracellular matrix components for tissue prototyping,^27^ and to build complex millimetric hexagonal 3D tissue architectures with multiple cellular PEGDA hydrogels.^28^ Arrays of hydrogel pads with variable stiffness polymerized in microfluidic channels^29^ were used to study cell behavior.^30^

The present contact lithography instrument enables to print well‐resolved microstructures with high reproducibility and flexibility, over a 23 mm circular area, with very short (<second) exposure times. As a proof‐of‐concept, a model experiment consisted of patterning thin monomer layers of controlled thickness (80–160 μm), sandwiched in between an acrylated glass slide and a PDMS‐coated slide. **Figure** **5** shows examples of resulting free‐standing structures polymerized on acrylated glass slides. High density arrays of wells with various shapes were successfully patterned (Figure S6, Supporting Information). Figure 5c shows 5 μm cells seeded in 60 μm PEG wells. Two‐layered structures were achieved by two successive polymerization cycles on the same substrate. Figure 5a shows a two‐layer microwell structure, with a functional bottom layer. A biotinylated bioprobe was immobilized in the bottom layer and could be selectively labeled with streptavidin–fluorophore conjugates later on.

**Figure 5:**
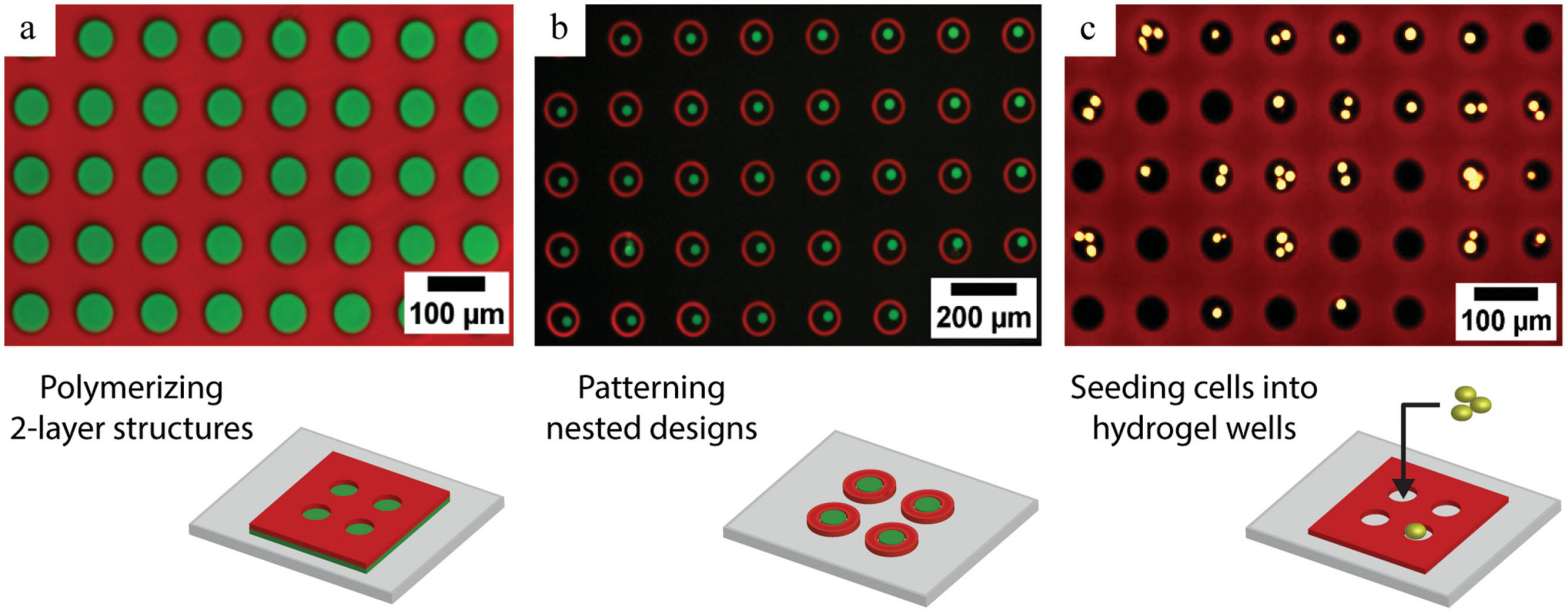
Complex multimaterial PEGDA structures patterned on surfaces (composite fluorescence images, 10× magnification). a) Two‐layered structure of 60 μm‐sized wells. A bottom sensing layer containing biotinylated oligonucleotides was grafted on the surface. Subsequently, a layer of hydrogel wells labeled with a red fluorophore was polymerized on top of the first layer. Later on, the hydrogel wells were incubated with a streptavidin‐AlexaFluor488 conjugate, which was captured by the biotin groups in the bottom layer. b) Nested designs. Posts (diameter: 20 μm, labeled with AlexaFluor488) and circles (diameter: 100 μm, labeled with AlexaFluor647) were sequentially polymerized on the surface. c) HSC‐3 cells labeled with calcein AM seeded in 50 μm diameter rhodamine‐labeled hydrogel wells.

Furthermore, while larger UV sources have been used by others to pattern microstructures across wide surface areas using lithography, a major added‐value of this set‐up is the possibility to precisely align masks and substrates with the top view live‐imaging module and precise motion controls. Circumventing the need for an expensive aligner, our system provides an affordable solution for efficient and quick prototyping of ­features larger than 10 μm. It is possible to pattern hydrogel structures at precise locations in microchannels for example. Additionally, multiple masks can be used sequentially to print complex interlocked structures from different materials. Figure 5b shows an example of such nested designs patterned with two different materials: 100 μm circular gels were polymerized around 20 μm posts (Figure S7, Supporting Information).

In this report, we have presented a cost‐efficient and versatile approach for the fabrication of both free‐floating polymer microparticles in microchannels and polymer microstructures on surfaces. High‐resolution UV‐induced polymerization of microstructures (20–150 μm) was successfully achieved using contact lithography across a 23 mm circular area, without the need for an expensive optical objective. By rationally designing a multichannel microfluidic chip for contact flow‐lithography, we were able to increase the synthesis rate for chemically homogenous particles by a 100‐fold in comparison to our standard microscope‐based technique. Contrary to replica molding and static contact lithography, flow lithography can be easily automated and operated as a continuous process. Millions of graphically encoded particles can be synthesized within an hour, with high particle reproducibility and resolution while avoiding clogging issues typical of particle suspensions at such high volume fractions. Additionally, this process can be applied to the fabrication of bit‐coded particles with extruded holes as well.

Finally, we believe that the reduced cost and portability represent a significant added value of the instrument. Indeed, there is a rising global interest in material science for solutions for scalable manufacturing and for equipment that enables accurate and efficient material fabrication at substantially lower overall cost.^31^ An increasing number of innovative, flexible, and open‐source designs are being reported in the literature.^32^ Built from scratch with an overall cost around $5000 (including the imaging unit), our system provides an efficient and versatile solution for particle and surface patterning, and easy prototyping, that can also easily be customized toward a specific application. In addition, the reduced cost, straightforward assembly, and small footprint enable parallelization of multiple polymerization stations and pave the path for particle production at industrial scale.

## Experimental Section

*Contact Flow Lithography Instrument*: A detailed part list can be found in the Supporting Information.

*Microfluidic Device Fabrication*: A microscope glass slide was spin‐coated with PDMS (200 μL, 3 min, 3000 rpm) and cured at 65 ºC for 30 min. The PDMS thick layer with channel imprints was fabricated through soft lithography on silicon wafers patterned with SU8. Inlet and outlet holes were punched using a 1.5 mm biopsy punch before assembling top and bottom layers. The device was baked at 65 ºC for 1 h, rinsed with ethanol, and dried with argon before use.

*Contact Flow Lithography*: A multichannel PDMS device was secured on the stage of the contact lithography instrument between slide holders. The PDMS device was connected to a pressured monomer reservoir and a collection vial using PTFE tubing (0.75 mm ID). The monomer reservoir consisted of a 1.5 mL Eppendorf microtube connected to a compressed air source (Tygon tubing 3/32 inch ID) using a 1.5 mL small reservoir microfluidic kit (Elveflow, France). Typical monomer composition for particle synthesis was PEGDA700 (20%), PEG600 (40%), Darocur 1173 (5%), and Tris‐EDTA 3X buffer (35%). To fabricate fluorescent particles, rhodamine‐B acrylate (Laysan‐Bio, USA) was added to the monomer solution. A 25 mm square chrome photomask with the desired shape pattern (Front range, CO, USA) was placed on the mask holder on top on the LED source. The photomask was aligned with the microchannels using the top view camera and alignment marks, and then brought up in contact with the device. Particles were polymerized and collected through sequential exposure (250 ms, 125 mW cm^−2^), flow, and stoppage steps. Detailed descriptions of the flow and illumination control systems can be found elsewhere.^8^, ^12^, ^17^ The collected particles were rinsed three times with aqueous buffer (Tris‐EDTA 1X buffer, 0.05% Tween20) using a 1:10 volume ratio to remove nonpolymerized monomer. Particles were imaged using bright field and/or fluorescence microscopy. Particle dimensions and fluorescence intensity were analyzed using ImageJ software (NIH, USA).

*Patterning Microstructures on Surfaces (Posts, Wells)*: Monomer solution was sandwiched between two thin glass slides, with double‐sided tape spacers (80 μm) used to control the height of the monomer layer. The bottom slide was acrylated beforehand to promote gel adhesion, whereas the top slide was spin‐coated glass with PDMS to prevent binding. Typical monomer composition for surface patterning synthesis was PEGDA700 (80%), Darocur 1173 (5%), Tris‐EDTA 3X buffer (15%). The device was placed on the instrument stage in contact with desired chrome photomask and exposed to UV light (typically 200 ms, 125 mW cm^−2^). Following exposure, the top slide was removed and the hydrogel layer was thoroughly rinsed with water to remove nonpolymerized monomer. When patterning multiple materials, the chamber was filled with the second monomer solution, sealed again with a PDMS‐coated slide, and aligned with a second photomask, before proceeding to the second polymerization run.

## Supporting information

As a service to our authors and readers, this journal provides supporting information supplied by the authors. Such materials are peer reviewed and may be re‐organized for online delivery, but are not copy‐edited or typeset. Technical support issues arising from supporting information (other than missing files) should be addressed to the authors.

Supplementary

Supplementary

Supplementary

Supplementary
