# Supplementary material for: High‐Throughput Contact Flow Lithography
Source: Adv Sci (Weinh). 2015 Jun 24;2(10):1500149. doi: 10.1002/advs.201500149 (PMC5115321; doi:10.1002/advs.201500149)
Supplement: Supplementary file 1 — Supplementary [file ADVS-2-0p-s003.pdf]

Copyright WILEY-VCH Verlag GmbH & Co. KGaA, 69469 Weinheim, Germany, 2015.

ADVANCED SCIENCE
Open Access

Supporting Information

for *Adv. Sci.*, DOI: 10.1002/advs.201500149

High-Throughput Contact Flow Lithography

Gaelle C. Le Goff, Jiseok Lee, Ankur Gupta, William Adam Hill, and Patrick S. Doyle**

Copyright WILEY-VCH Verlag GmbH & Co. KGaA, 69469 Weinheim, Germany, 2013.

Supporting Information

High-throughput contact flow lithography

Gaelle C. Le Goff[†], *Jiseok Lee*[†], *Ankur Gupta, W. Adam Hill*, and Patrick S. Doyle**

[†] These authors contributed equally to this work.

Dr. G. C. Le Goff
Department of Chemical Engineering, Massachusetts Institute of Technology, 77 Massachusetts Avenue, Cambridge, MA 02139, USA

Novartis Institutes for Biomedical Research, 250 Massachusetts Avenue, Cambridge MA 02139, USA

Dr. J. Lee
Department of Chemical Engineering, Massachusetts Institute of Technology, 77 Massachusetts Avenue, Cambridge, MA 02139, USA

School of Energy and Chemical Engineering, Ulsan National Institute of Science and Technology, Eonyang-eup, Ulju-gun, Ulsan 689-798, Korea

Dr. W. A. Hill
Novartis Institutes for Biomedical Research, 250 Massachusetts Avenue, Cambridge MA 02139, USA
E-mail: adam.hill@novartis.com

A. Gupta, Prof. P. S. Doyle
Department of Chemical Engineering, Massachusetts Institute of Technology, 77 Massachusetts Avenue, Cambridge, MA 02139, USA
E-mail: pdoyle@mit.edu

Contents

1. Contact flow lithography instrument 2
2. Design of multichannel device for stop-flow contact lithography 4
3. Stop-flow contact lithography: Results 8
4. Micro-structure patterning 10

1. Contact flow lithography instrument

Contact flow lithography instrument part list. 365 nm UV LED light and white light sources, aspheric condenser lens, CMOS camera, dichroic cage cube, dichroic mirror, filter holder, Ø1" lens tubes, XY Translator, 30 mm cage, damped posts, bread board, right-angle bracket translation stages, stage for microfluidic device (60 mm Cage Mount for Cylindrical Lenses) and cage post (Thorlabs Inc.); 4X Plan Achromat objective 0.10NA 18.5 mmWD (Olympus); XYZ-rotation sample stage (Suruga Seiki); custom 3D printed mask holder.

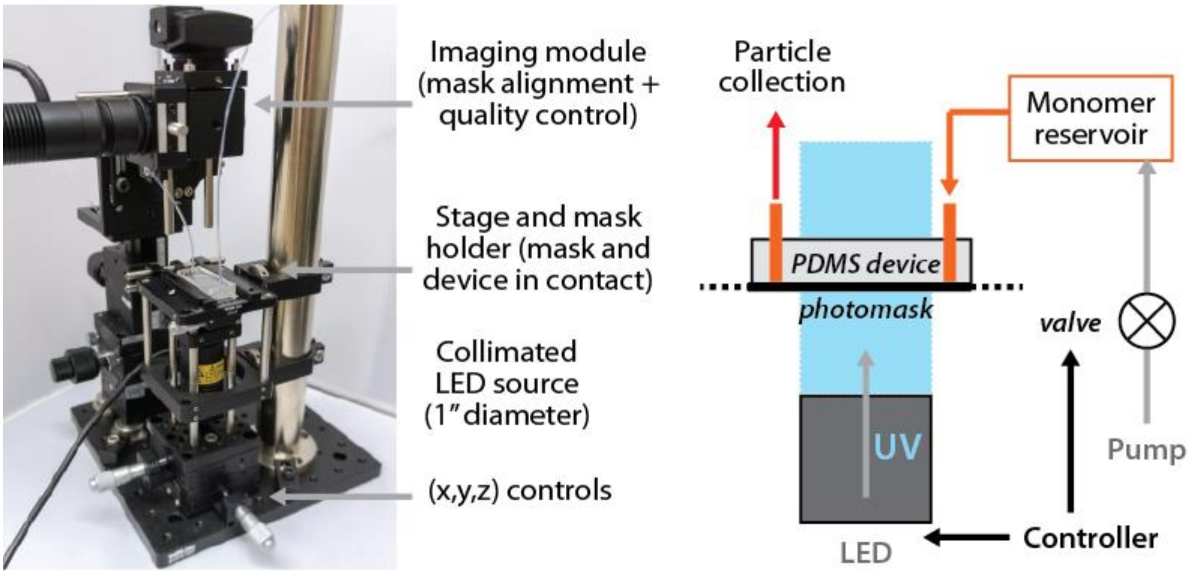

Figure S1. Contact flow lithography instrument and scheme of stop-flow contact lithography process.

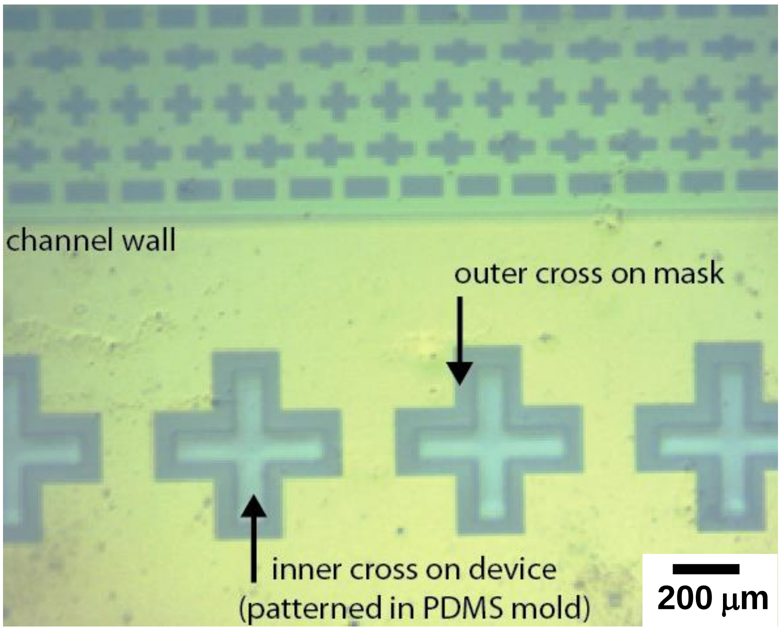

Figure S2. Alignment of photomask and channel (view from the instrument camera). Matching alignment marks are included in the photomask and PDMS device designs. A blue dye was used to color the monomer solution.

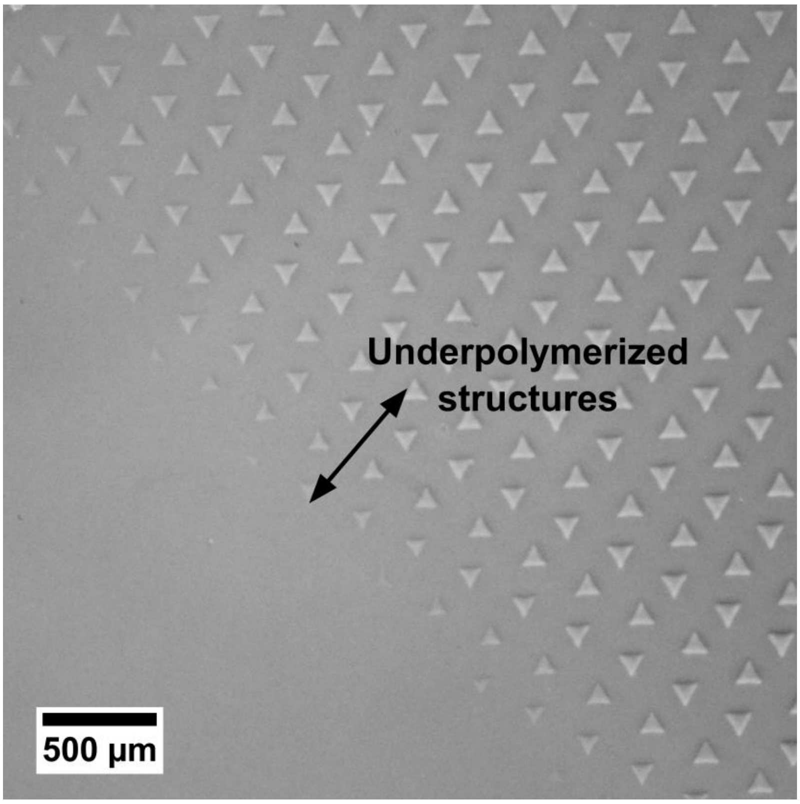

Figure S3. Brightfield image of PEG microstructures polymerized on a glass substrate at the edge of the UV LED beam. Particle quality decreases only in a 1-mm zone at the border of the 25-mm beam.

2. Design of multichannel device for stop-flow contact lithography

The design of the multichannel device for stop-flow lithography was optimized in two steps. First, a single straight channel was considered as a model to assess the impact of the device dimensions on the particle synthesis rate. It should be noted though, that this analysis does take into account neither the resistance arising from particles present in the channel, nor the interaction between those particles and the channel top and bottom walls. Second, simulations were run to optimize the layout of the multiple channels.

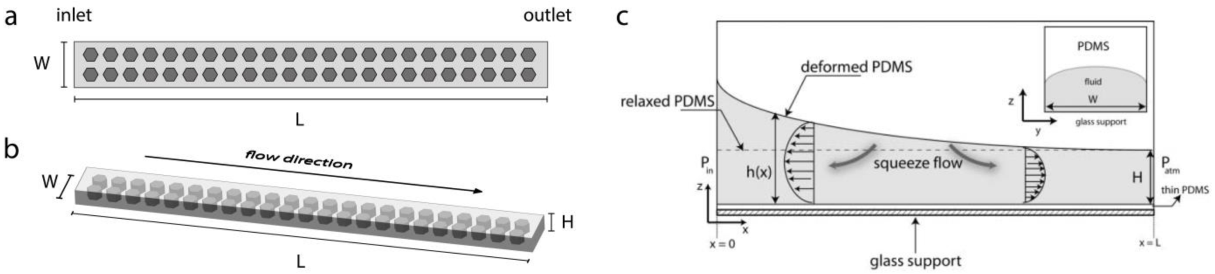

Figure S4. Dimensions of the PDMS microfluidic channel. (a) Top and (b) side view of the microfluidic unit channel filled with particles (L=length; W=width; H=height). (c) Deformation of PDMS channel under pressure; reproduced from reference.[1]

1. Rate of particle synthesis in a straight PDMS channel

Let's consider a PDMS straight channel bearing a rectangular cross-section as depicted in **Figure S4 (a, b)** with L>>H and W>>H.

Channel deformation

The device top layer is a PDMS deformable layer (Young's modulus, E ~ 1 MPa)[2] of a few millimeters. Due to PDMS elasticity, the channel slightly deforms when pressure is applied at the inlet, locally increasing its cross-section (**Figure S3c**). The deformation of the very thin PDMS bottom layer, however, can be neglected as it is spin-coated on a rigid glass slide (E = 62 GPa). A detailed analysis of coupling of flow and elasticity for the creeping flow (Re << 1) of fluid through a thin PDMS channel can be found in a previous report by

Dendukuri et al. (H/L << 1, no-slip boundary conditions imposed at the top and bottom walls).[1] The authors demonstrate that the maximum deformation Δh_{max} occurs at the channel entrance and is given by **Equation S1** for W/H>>1 (P = inlet pressure).

$$\Delta h_{max} \sim \frac{PW}{E} \quad (S1)$$

Rate of particle synthesis

The rate of particle synthesis can be defined as the ratio of the number of particles synthetized per exposure (n_p) to the cycle duration (t_{cycle}) (**Equation S2**). The number of particles per exposure n_p is proportional to the channel width and length. The cycle duration can be decomposed in three contributions: t_{pol}, t_{flow} and t_{stop}.

$$rate = \frac{n_p}{t_{cycle}} \sim \frac{WL}{t_{pol} + t_{flow} + t_{stop}} \quad (S2)$$

Polymerization time (t_{pol}): corresponds to the UV exposure (~0.2 s) followed by a short hold period (~0.2 s) in the steady fluid for complete polymerization. This parameter is independent from the device dimensions and usually negligible.

Flow time (t_{flow}): is the minimum time required to flush all particles out of the channel. It corresponds to the time required for a particle to travel from the inlet to the outlet of the channel. The expression of t_{flow} for a rigid rectangular channel is given by **Equation S3**, where ν is the flow velocity, Q the hydrodynamic resistance of the channel, and μ the viscosity of the monomer. The rigid channel model slightly overestimates the flow time. Indeed, taking into account the PDMS elasticity would result in lower flow times, as the PDMS deformation lowers the channel resistance.

$$t_{flow} = \frac{L}{v} = \frac{L}{Q/LW} \sim \frac{12\mu L^2}{\Delta P H^2} \quad (S3)$$

Stoppage time (t_{stop}): is the device relaxation time after releasing the pressure constrain is released at the inlet. Considering the coupling of flow and elasticity, Dendukuri et al.

demonstrated the scaling law given in **Equation S4** for the device for t_{stop}.[1] ΔPW/E characterizes the deformation of the channel.

$$t_{stop} \sim \frac{4\mu L^2 W}{EH^2}\left(\frac{1}{\frac{\Delta PW}{E}+\frac{H}{3}}\right) \quad (S4)$$

The resulting scaling law for the synthesis rate is given in **Equation S5**.

$$rate \sim \frac{1}{\frac{4\mu L}{EH^2}\left(\frac{1}{\frac{\Delta PW}{E}+\frac{H}{3}}+\frac{3E}{\Delta PW}\right)} \quad (S5)$$

Influence of channel parameters

According to Equation S5, increasing the channel width or height leads to an increase in synthesis rate. We chose a channel height H of 50 μm in order to produce particles with 45 μm in height The W/H aspect ratio was limited by fabrication constraints. Indeed, for W/H > 20 (W > 1 mm), the top wall of the PDMS channel sags. The selected width was 950 μm.

Equation S1 can be used to calculate the pressure for which the deformation of the channel at the inlet is equivalent to the height of the channel with H = 50 μm and W = 950 μm.

$$\Delta P \sim \frac{HE}{W} \sim 7.6\, psi \quad (S6)$$

With all other parameters fixed, **Equation S5** shows that increasing in the channel length tends to decrease the rate of particle synthesis. Indeed, longer channels increase both the particle flushing time and the flow stoppage time. Therefore, designs involving multiple short channels are preferable.

References

[1] D. Dendukuri, S. S. Gu, D. C. Pregibon, T. A. Hatton, P. S. Doyle, *Lab Chip* **2007**, *7,* 818.

[2] T. Gervais, J. El-Ali, A. Gunther, K. F. Jensen, *Lab Chip* **2006**, *6,* 500.

2. Simulations

Eight parallel channels were coupled in a multichannel design with a common inlet and outlet. Two of these modules can be run in parallel, covering a 16 mm per 10 mm polymerization zone. Quasi-2D hydrodynamic simulations were used to select a splitting that ensures homogenous flow rates across the channels (**Figure S5**).

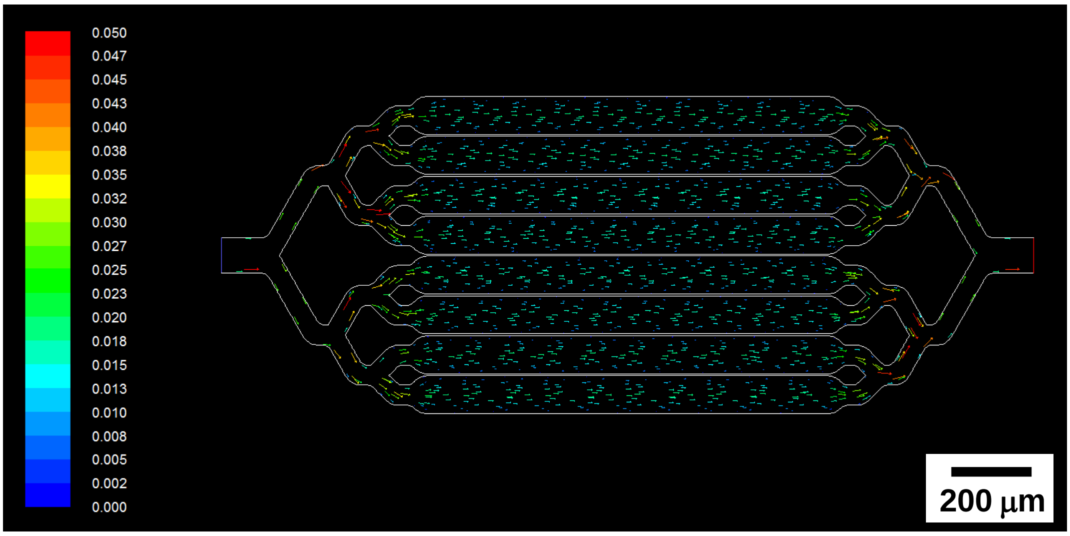

Figure S5. Velocity vector map in m s^{-1} (quasi-2D simulation, inlet velocity set at 0.01 m s^{-1})

3. Stop-flow contact lithography: Results

Movies

Movie S1 shows ten consecutive synthesis cycles of particles with various shapes (~ 75 μm) using a polyethylene glycol diacrylate (PEGDA) monomer (blue food coloring dye was added to the monomer solution to enhance contrast). The parameters used for the synthesis cycle were the following: pressure 8 psi, t_{pol} 0.5 s (0.25 s UV exposure + 0.25 s hold period), t_{flow} 5 s and, t_{stop} 2.5 s.

Movie S2 demonstrates the synthesis of square particles (75 μm x 75 μm) using a high density photomask (polyethylene glycol diacrylate monomer with blue food coloring dye added to the monomer solution to enhance contrast).

Movie S3 demonstrates the synthesis of triangle particles (~ 75 μm) using a high density photomask and polyurethane acrylate as a monomer.

Table S1. Particle reproducibility. Rhodamine-B labeled fluorescent PEGDA particles were synthetized using photomask with four different feature shapes. The characteristic dimensions and thickness of particles were in excellent agreement with the photomask and channel dimensions, with coefficients of variation (CV) lower than 4% for all particle types.

Particle shape and characteristic length		Median [μm]	SD [μm]	CV [%]	Patterning dimension [μm]
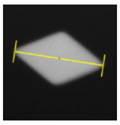	diamond length	136.2	2.6	1.9 (n=30)	139[a]
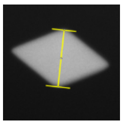	diamond width	79.9	1.6	2.1 (n=30)	80[a]
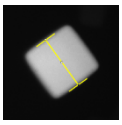	square edge	80.1	1.9	2.4 (n=31)	75[a]
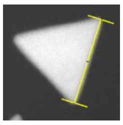	triangle edge	112.2	4.0	3.6 (n=31)	114[a]
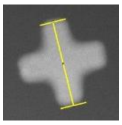	cross length	108.2	1.9	1.8 (n=36)	100[a]
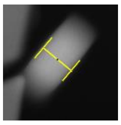	particle thickness	44.8	1.5	3.3 (n=12)	45[b]

[a] Feature dimension on chrome photomask
[b] Expected particle thickness for polymerization in a 50 μm-thick PDMS channel (inhibition layer ~ 2.5 μm)

4. Micro-structure patterning

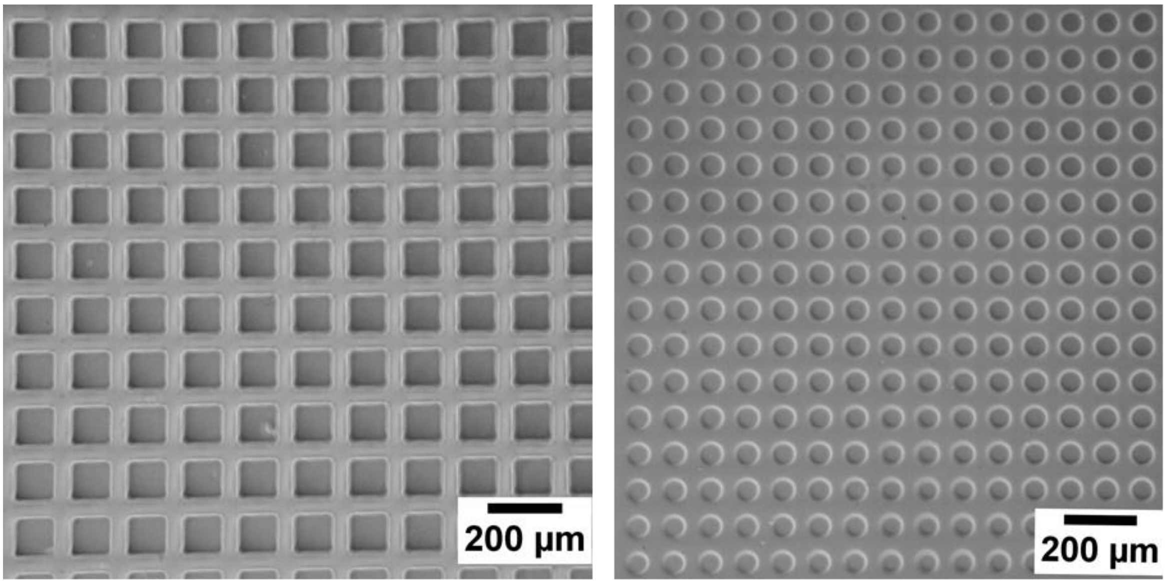

Figure S6. Bright field image of arrays of (left) square or (right) rounded PEGDA microwells polymerized on a glass surface. Well depth is 80 μm.

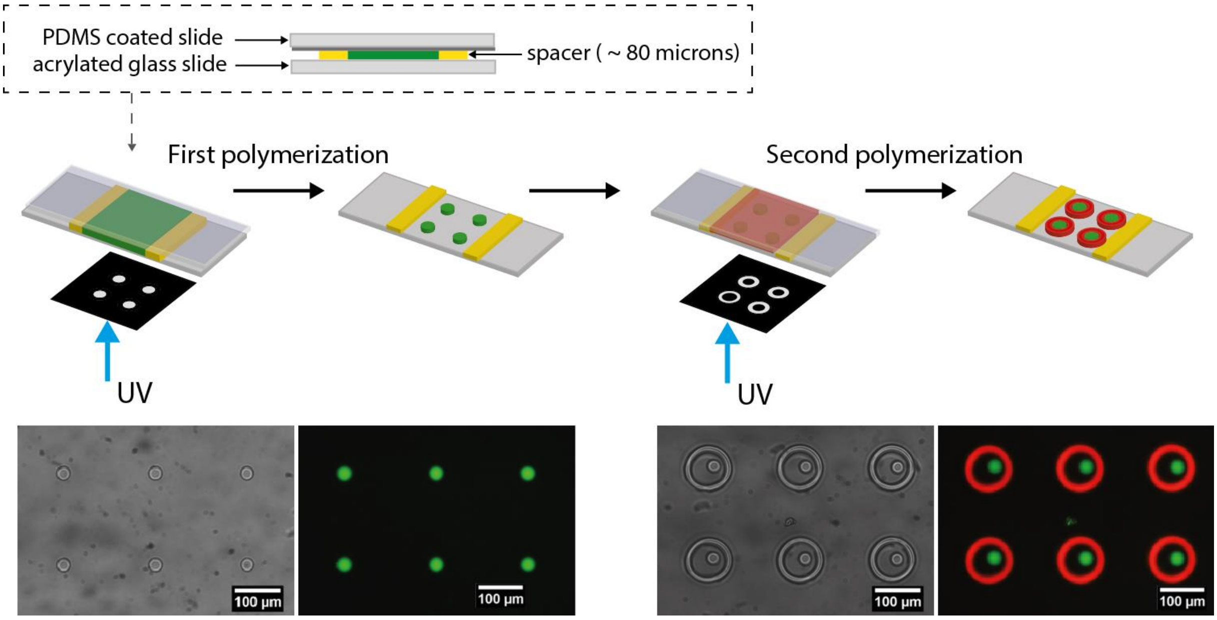

Figure S7. Patterning nested PEGDA microstructures using two monomer compositions (including different fluorophores): (top) workflow and (bottom) bright field and fluorescence images.
